# Alzheimer's disease pathology is associated with earlier alterations to blood–brain barrier water permeability compared with healthy ageing in TgF344‐AD rats

**DOI:** 10.1002/nbm.4510

**Published:** 2021-03-15

**Authors:** Ben R. Dickie, Hervé Boutin, Geoff J. M. Parker, Laura M. Parkes

**Affiliations:** ^1^ Division of Neuroscience and Experimental Psychology, Faculty of Biology, Medicine, and Health, Stopford Building University of Manchester Manchester UK; ^2^ Geoffrey Jefferson Brain Research Centre Manchester Academic Health Science Centre Manchester UK; ^3^ Wolfson Molecular Imaging Centre, Faculty of Biology, Medicine, and Health University of Manchester Manchester UK; ^4^ Bioxydyn Ltd Manchester UK; ^5^ Centre for Medical Image Computing, Department of Computer Science and Department of Neuroinflammation University College London London UK

**Keywords:** ageing, Alzheimer's disease, BBB breakdown, BBB dysfunction, BBB permeability, blood–brain barrier, MRI, TgF344‐AD

## Abstract

The effects of Alzheimer's disease (AD) and ageing on blood–brain barrier (BBB) breakdown are investigated in TgF344‐AD and wild‐type rats aged 13, 18 and 21 months. Permeability surface area products of the BBB to water (*PS*_w_) and gadolinium‐based contrast agent (*PS*_g_) were measured in grey matter using multiflip angle multiecho dynamic contrast‐enhanced MRI. At 13 months of age, there was no significant difference in *PS*_w_ between TgF344‐AD and wild‐types (*p* = 0.82). Between 13 and 18 months, *PS*_w_ increased in TgF344‐AD rats (*p* = 0.027), but not in wild‐types (*p* = 0.99), leading to significantly higher *PS*_w_ in TgF344‐AD rats at 18 months, as previously reported (*p* = 0.012). Between 18 and 21 months, *PS*_w_ values increased in wild‐types (*p* = 0.050), but not in TgF344‐AD rats (*p* = 0.50). These results indicate that BBB water permeability is affected by both AD pathology and ageing, but that changes occur earlier in the presence of AD pathology. There were no significant genotype or ageing effects on *PS*_g_ (*p* > 0.05). In conclusion, we detected increases in BBB water permeability with age in TgF344‐AD and wild‐type rats, and found that changes occurred at an earlier age in rats with AD pathology.

## Graphical abstract

In this study we use multi‐flip angle multiecho dynamic contrast‐enhanced MRI to measure BBB surface area products of gadolinium contrast agent (*PS*_g_) and water (*PS*_w_) in TgF344‐AD rats and wild‐types at multiple time points to assess ageing effects. We observe significant increases in *PS*_w_ with age in both TgF344‐AD and wild‐type rats but found changes occurred earlier in TgF344‐AD rats. No alterations in *PS*_g_ were observed with age or genotype.

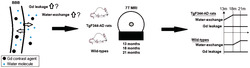

**Abbreviations used**
AD: Alzheimer's disease
BBB: blood–brain barrier
CoV: coefficient of variation
MFAME‐DCE MRI: multi‐flipangle multiecho dynamic contrast‐enhanced MRI
*PS*_g_
: permeability surface area product of the blood–brain barrier to gadolinium‐based contrast agent
*PS*_w_
: permeability surface area product of the blood–brain barrier to water
ROI: region of interest
SNR: signal‐to‐noise ratio
SPGR: spoiled gradient echo
SSS: superior sagittal sinus

## 1 INTRODUCTION

Gathering evidence now supports age‐related blood–brain barrier (BBB) breakdown,^1^, ^2^, ^3^ particularly in the hippocampus,^4^ a region commonly associated with early pathogenesis of Alzheimer's disease (AD). The BBB is further impaired in patients with early and established AD,^5^, ^6^ including increased blood–brain leakage of blood‐derived proteins in the hippocampus and cortex,^7^, ^8^ altered expression of BBB amyloid‐β transporters in the hippocampus,^9^, ^10^, ^11^ loss of pericytes,^4^, ^12^ decreased expression of tight junction proteins in the hippocampus and cortex,^13^, ^14^, ^15^ blocked or dysfunctional interstitial drainage pathways^16^, ^17^ and altered astrocytic aquaporin‐4 expression.^18^ A small number of *in‐vivo* tracer studies also support the presence of BBB breakdown, showing increased BBB permeation of MRI contrast agents in the hippocampus and cortex.^4^, ^12^, ^19^ The impact of BBB dysfunction on brain health is increasingly recognised. Bowman et al. showed that BBB breakdown measured using the cerebrospinal fluid (CSF) albumin index was linked to cognitive abilities in healthy older adults,^20^ and Nation et al. showed that BBB breakdown as measured using dynamic contrast‐enhanced MRI was associated with cognition in patients with early AD, independent of amyloid‐β, tau and vascular risk status.^12^

Despite many reports of BBB alterations in AD, the timescales of these changes, and how they differ compared with age‐related BBB alterations, are poorly understood. Rodent models of AD allow studies spanning the equivalent of approximately 20 human years from prodromal to advanced AD to be performed in a much shorter timeframe (approximately 1–2 years) and enable assessment of purely AD and age‐related changes in the absence of vascular risk factors. In our previous work, we demonstrated that TgF344‐AD rats aged 18 months exhibit higher BBB water permeability relative to wild‐types (WTs), which correlated with loss of the tight junction protein occludin‐1. We also found that these changes did not affect the leakage rate of gadolinium‐based contrast agent,^15^ indicating that BBB impairment due to the AD genotype was subtle.

In this study, we assess age‐ and AD‐related BBB breakdown in TgF344‐AD and WT rats using MRI at two additional time points (13 and 21 months) to aid understanding of the time course of AD‐related changes with reference to the normal age‐related trajectory. In statistical analyses, we combine this data with our previously published 18‐month data, which was acquired using the same imaging protocol in a different colony of TgF344‐AD rats. We used multi‐ fllipangle multiecho dynamic contrast‐enhanced (MFAME‐DCE) MRI to noninvasively measure the BBB permeability surface area products of water (*PS*_w_) and gadolinium‐based contrast agent (*PS*_*g*_) to simultaneously study various degrees of BBB alterations.

## 2 MATERIALS AND METHODS

### 2.1 Animals

Two male and two female WT Fischer and TgF344‐AD rats with the APP_swe_ and PS1_Δe9_ mutations were purchased from the laboratory of Prof T. Town (University of Southern California) and were set up as breeding pairs, housed in the Biological Services Unit at the University of Manchester. Genotyping was outsourced to Transnetyx. Experimental procedures were approved by the Preclinical Imaging Executive Committee of the University of Manchester and carried out in accordance with the UK Animals (Scientific Procedures) Act 1986 and EU Directive 2010/63/EU for animal experiments. Breeding, housing and husbandry details, as recommended by the ARRIVE guidelines,^21^ can be found in the supporting information.

Rats aged 13.3 ± 0.6 months (13 TgF344‐AD [four females {F}: nine males {M}]; 16 WT [10 F: six M]) and 21.3 ± 1.5 months (eight TgF344‐AD [three F: five M]; seven WT [four F: three M]) were scanned under anaesthesia (4% isoflurane for induction followed by 2.5% isoflurane for maintenance in 100% O_2_ at 1 L/min). To evaluate the effects of age on *PS*_w_ and *PS*_g_ more thoroughly, previously published MRI data from 18‐month‐old rats from a different cohort were included in statistical analyses (seven TgF344‐AD and five WT; all male).^15^ Animals from both cohorts were bred and kept under identical conditions, and the MRI protocols and analyses were identical. Of the 29 TgF344‐AD and WT rats scanned at 13 months, 14 were rescanned at 21 months. Eight rats scanned at 13 months were scanned twice within 2 weeks to assess the scan‐rescan repeatability of MRI measures. The attrition of rats between the 13 and 21‐month time points was because 14 rats were culled after the 13‐month time point for ex‐vivo analyses (for further comments, see the Discussion section), not because of natural or disease‐related deaths.

### 2.2 MRI

MFAME‐DCE MRI was used to measure the BBB permeability surface area products to water (*PS*_w_) and a small molecular weight gadolinium‐based contrast agent (*PS*_*g*_). MRI scans were acquired on a Bruker Avance III console interfaced with an Agilant 7T 16‐cm bore magnet. A Bruker transmit‐only resonator (T11070V3) was used for transmission and a Bruker rat brain surface coil (T11205V3) was used for signal reception. A high‐resolution *T*_2_‐RARE anatomic volume was acquired for the purpose of region of interest (ROI) segmentation, as described later. The scan parameters were: TR/TE = 3188/11 ms, NEX = 2, voxel size = 0.12 × 0.12 × 1 mm^3^, and matrix size = 256 × 256 × 64. Native *T*_1_ was measured using coronal variable flip angle 3D spoiled gradient echo (SPGR) scans with the following acquisition parameters: α = 10°, 20°, 40° and 60°; TR/TE = 100/2.1 ms, voxel size = 0.46 × 0.46 × 0.31 mm^3^, and matrix size: 64 × 64 × 96. *T*_1_ estimates were corrected for *B*_1_ inhomogeneity by jointly fitting to volumes acquired with short and long TR, as previously reported.^15^, ^22^ Coronal dynamic 3D SPGR volumes were acquired at a single flip angle before and during intravenous injection of gadoteric acid (Dotarem, Guerbet). A dose of 0.5 mmol kg^−1^ was delivered though a 24G catheter with an electronic pump at 1 mL min^−1^. The acquisition parameters for these volumes were: 15 volumes, α = 60°; TR/TE = 20/2.1 ms, voxel size = 0.46 × 0.46 × 0.31 mm^3^, and matrix size: 64 × 64 × 96. Following these scans, coronal multiecho 3D SPGR volumes were acquired at multiple flip angles. The flip angle was varied to alter the sensitivity of MRI signals to BBB water‐exchange to enable estimation of *PS*_w_, as previously reported.^15^ The acquisition parameters for these volumes were: 6 volumes per flip angle, α = 30°, 40°, 20°, 10° and 80°; TR = 100 ms, ΔTE = 2.14 ms, 10 echoes, voxel size = 1 × 1 × 1 mm^3^, and matrix size: 32 × 32 × 30. To conclude the scan, a final set of five single‐echo single flip angle SPGR volumes were acquired with the same acquisition parameters as the first 15 dynamic volumes. The slab select direction was placed along the superior–inferior direction to ensure nonselective excitation of magnetisation along the rostral‐caudal direction to minimise *T*_1_‐related inflow effects.

### 2.3 MRI analysis

MRI data were analysed at an ROI level to maximise signal‐to‐noise ratio (SNR). The Schwarz et al. atlas^23^ was used to define ROIs for the hippocampus, cortex, thalamus and striatum by pooling together smaller substructures contained within (see Table 1 for details of the substructures included in each ROI). ROIs were extracted for each rat by registering the reference image from the Schwarz et al. rat brain atlas to the high‐resolution *T*_2_‐RARE image.^23^ This was done using the Insight Toolkit within the Advanced Normalisation Tools package. The associated label image was then downsampled to the spatial resolution of the SPGR volumes. Voxels containing significant CSF were excluded from ROIs using a mask calculated by thresholding the precontrast *T*_1_ maps at *T*_1_ less than 3 s.

**TABLE 1:** Scan‐rescan coefficient of variation (%) for *PS*_w_ and *PS*_g_, alongside a description of the regions of interest (ROIs) including their mean size ± sd (number of voxels), mean volume ± sd (mm^3^), and the substructures used for pooling

ROI details				Scan‐rescan coefficient of variation (%)	
ROI name	Size (voxels)	Volume (mm^3^)	Pooled substructures	*PS*_*w*_	*PS*_*g*_
Composite ROI	7013 ± 855	480 ± 58	Hippocampus, cortex, striatum and thalamus	14	8
Hippocampus	1476 ± 144	101 ± 9.8	Antero‐dorsal, posterior, subiculum and ventral	23	13
Cortex	3452 ± 510	236 ± 35	Cingulate, entorhinal, frontal association, medial prefrontal, parietal association, somatosensory, retrosplinal and temporal association cortices	38	23
Striatum	1246 ± 175	85 ± 12	Caudate putamen, globus pallidus	13	12
Thalamus	839 ± 141	57 ± 10	Dorsolateral, midline dorsal, ventromedial	14	19

**FIGURE 1:**
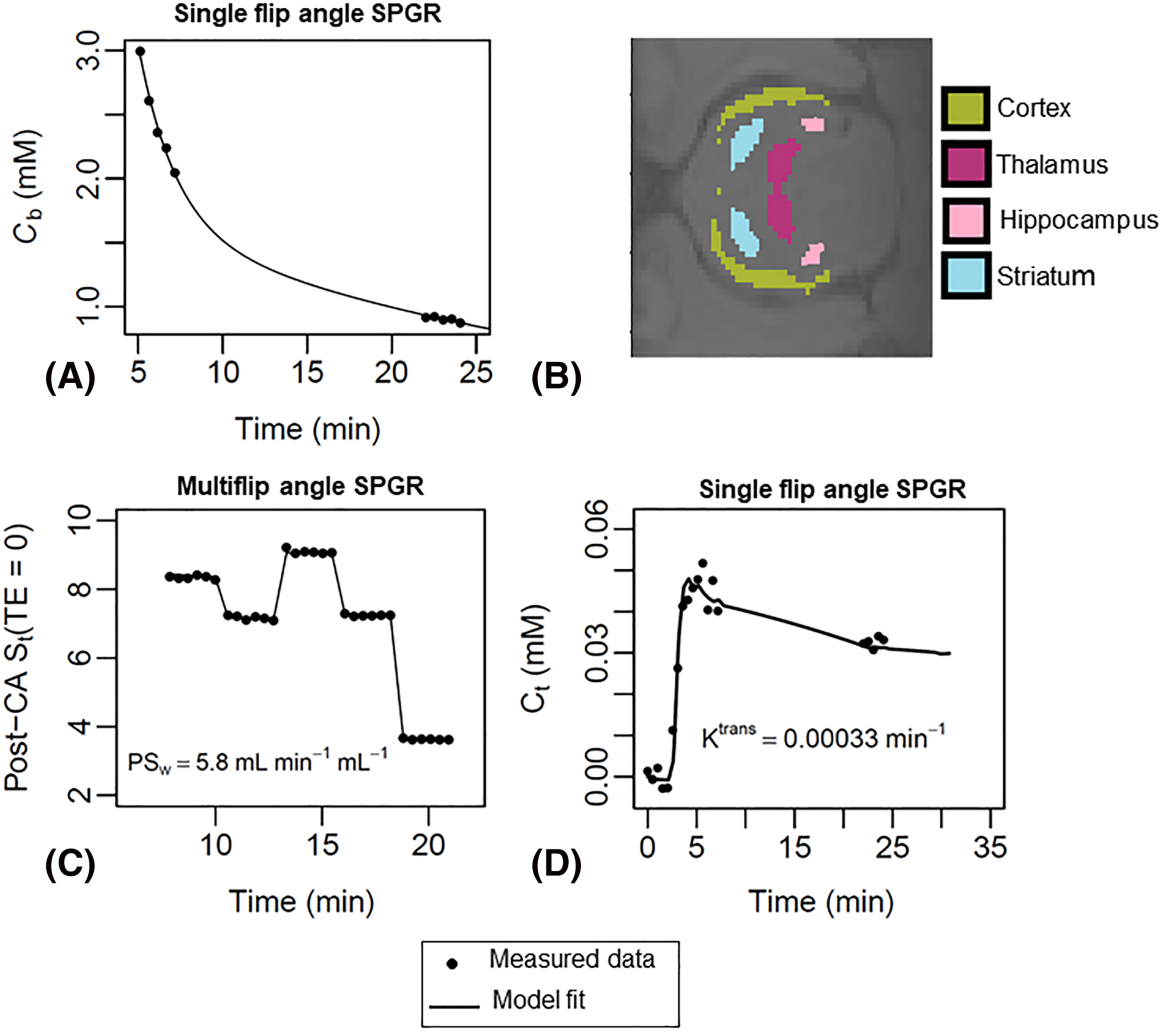
Example superior sagittal sinus (SSS) vascular input function (VIF), regions of interest (ROIs) and model fits to MFAME‐DCE MRI data. (A) Example *C*_b_(t) estimate and the corresponding biexponential fit used to define the VIF from the SSS. The biexponential fit was used to infer blood contrast agent concentration during acquisition of multiflip angle data. The procedure of extracting a VIF was performed individually for each rat to capture interindividual variability in cardiac output, renal clearance and total blood volume. (B) Example segmentations of hippocampus, cortex, striatum and thalamus ROIs. These ROIs were combined to form a composite ROI for statistical analyses; (C) example two‐site water‐exchange model fits to multiflip angle data yielding estimates of *PS*_w_; and (D) example Patlak model fits to single flip angle data yielding estimates of *PS*_g_. SPGR, spoiled gradient echo

For each rat, the four individual ROIs were averaged to form a composite grey matter ROI. Median MRI signals for the composite ROI, and each individual ROI, were then extracted for model fitting. MRI signal and precontrast *T*_1_ in blood were extracted from the superior sagittal sinus (SSS) for calculation of the blood concentration time course *C*_b_(t), also known as the vascular input function (VIF). The SSS ROI was defined as follows: a slice containing the SSS was manually selected from the fourth postcontrast volume (SSS appears bright). A histogram of decay‐corrected signals from this slice was generated and voxels with signal in the 99th percentile from all voxels in the slice were selected to be included in the ROI. Quality control checks were performed to ensure these voxels did indeed arise from the SSS, and not from other vessels in the brain. During the multiple flip angle scans, the VIF was not measured directly, but inferred from a biexponential fit to *C*_b_(t) measured from the single flip angle data (Figure 1A).

To estimate *PS*_*g*_ [mL min^−1^ mL^−1^ ], the Patlak model was fit to the single flip angle data, assuming fast BBB water‐exchange (*PS*_w_ = ∞).^24^ In the case of low‐level BBB impairment, transfer of small molecular weight gadolinium‐based MRI contrast agents across the BBB are permeability‐limited and *PS*_*g*_ is equivalent to the contrast agent volume transfer constant *K* ^trans^.^25^ To estimate *PS*_w_ (mL min^−1^ mL^−1^), data with multiple flip angles were first corrected for *T*_2_* decay by fitting an exponential decay model, leading to estimates of S(TE = 0). A two‐site water‐exchange model described previously^15^ was then fit to estimates of S(TE = 0), assuming *PS*_*g*_ = 0 mL min^−1^ mL^−1^. Our previous work^15^ shows that bias introduced into estimates of *PS*_*w*_ by assuming *PS*_*g*_ = 0 is small for *PS*_*g*_ < 10^−3^ mL min^−1^ mL^−1^. All model parameters were estimated by minimising the sum of squared residuals between the model and data using a Levenberg–Marquart optimisation algorithm in R (version 4.0.2). No blinding to genotype or age was performed. Full details of MFAME‐DCE MRI acquisition and analysis are described in Dickie et al.^15^

### 2.4 Scan‐rescan repeatability and estimation of within‐group biological variability

Scan‐rescan repeatability for composite and regional *PS*_w_ and *PS*_*g*_ estimates was calculated from 13‐month scan‐rescan data by computing the coefficient of variation (CoV) of repeats using the root mean square method^26^:

$$\mathrm{Co}V_{R}=\sqrt{\frac{1}{2n}\sum {\left(\frac{d_{i}}{m_{i}}\right)}^{2}}$$

where *d*_i_ are the differences between paired measurements, *m*_i_ are the means of the paired measurements and *n* (= 8) is the number of pairs of measurements. CoV estimates are given in Table 1.

Correlation and Bland–Altman analysis was performed across all regional estimates of *PS*_w_ and *PS*_*g*_ to determine the coefficient of determination (*R* ^2^) and limits of agreement between repeat measures.

An estimate of variability in *PS*_w_ and *PS*_*g*_ attributable to biological differences between rats (*CoV*_*B*_) was calculated from scan 1 repeatability data by assuming biological variance and variance associated with random measurement error add‐in quadrature to equal the total within‐group variance ( $\mathrm{Co}V_{W}^{2})$:

$$\mathrm{Co}V_{B}^{2}=\mathrm{Co}V_{W}^{2}-\mathrm{Co}V_{R}^{2}$$

*CoV*_*W*_ was estimated by computing the standard deviation of scan 1 measurements divided by the mean of these same measures.

### 2.5 Statistical analysis

All statistical analyses were performed in R (version 4.0.2). Prior to statistical analysis, outliers greater than ± 2 standard deviations from group means were removed (resulting in removal of approximately 6% of all data points), and tests for normality on the resulting distributions were performed using Shapiro–Wilk tests. The null hypothesis that data was normally distributed was rejected in one of 12 groups (*PS*_w_ in 13‐month‐old WTs; *p* = 0.003). Because most groups (11/12) displayed normally distributed data, parametric tests were used in further analyses.

The effects of AD and age on composite ROI *PS*_w_ and *PS*_g_ were assessed using a mixed‐effects model (lme function in R) with random effect of subject, and fixed effects of age, genotype and the age × genotype interaction. We did not model the effect of brain region on *PS*_w_ and *PS*_g_ as the study was not powered to detect region‐level effects, especially considering that these may vary with age. Because AD and ageing are known to preferentially affect hippocampal BBB integrity, effects on hippocampal *PS*_w_ and *PS*_g_ were investigated as an exploratory analysis. The mixed‐effects model used for both analyses is appropriate for data with partial repeated measures (i.e. because some but not all 13‐month‐old animals had repeat scans at 21 months, and 18‐month‐old rats were scanned only once). Heteroskedastic errors in levels of the age factor were modelled to account for differences in measurement variance across age. Tukey post hoc tests were used to assess pairwise effects.

To investigate whether measurements of *PS*_w_ and *PS*_g_ were related, linear regression analysis was performed. Separate regression lines were fit for each age group.

## 3 RESULTS

**FIGURE 2:**
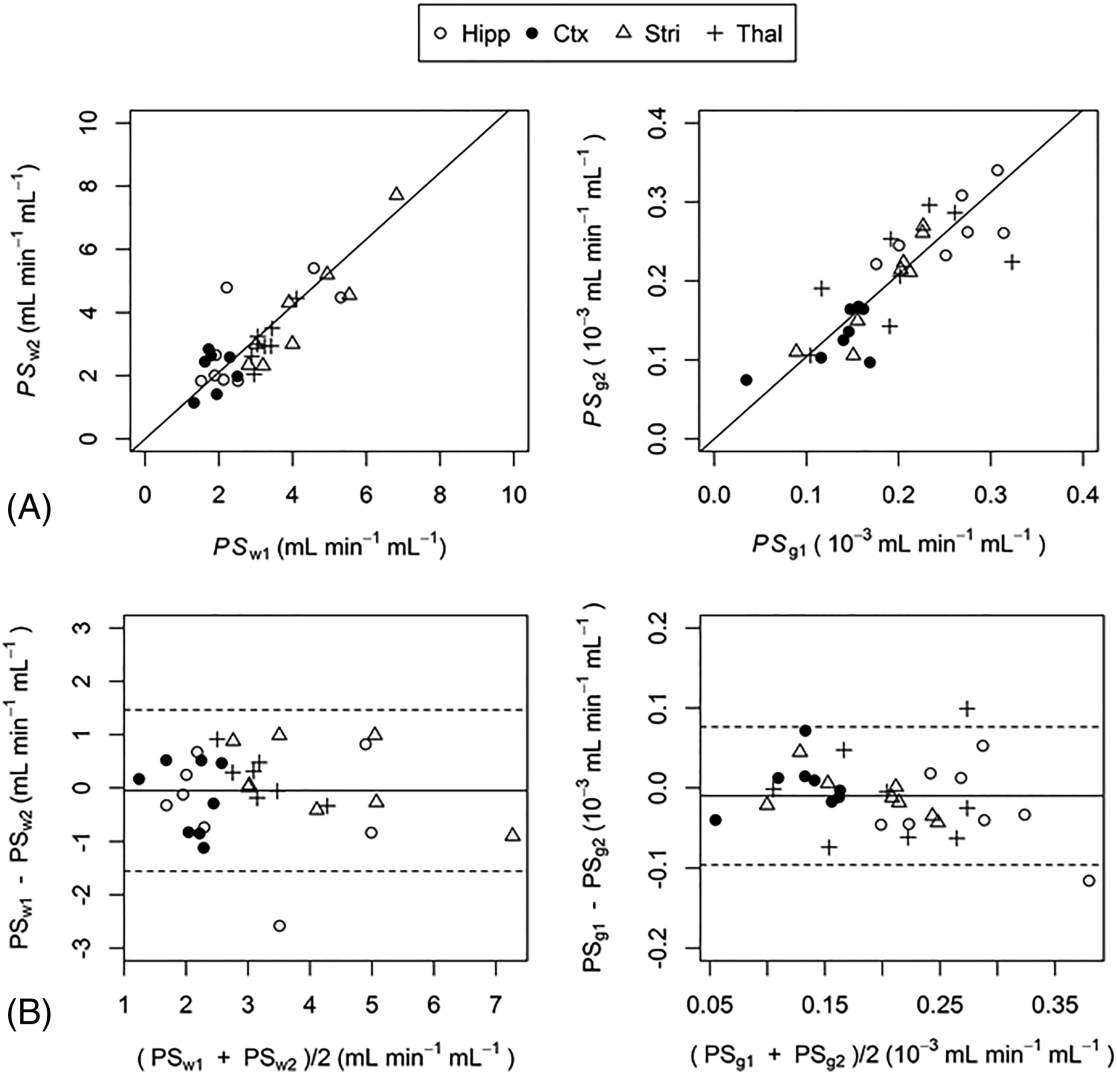
Correlation (A) and Bland–Altman (B) plots showing the agreement between regional scan‐rescan measurements of *PS*_w_ and *PS*_g_ *. PS*_w_ and *PS*_g_ had coefficient of determination values (*R* ^2^) of 0.82 (*p* < 10^−12^) and 0.96 (*p* < 10^−16^), respectively. Solid lines in the Bland‐Altman plots show the mean difference between scan 1 and scan 2. Dashed lines show the limits of agreement within which 95% of scan‐rescan differences lie. Hipp, hippocampus; Ctx, cortex; Stri, striatum; Thal, thalamus

**FIGURE 3:**
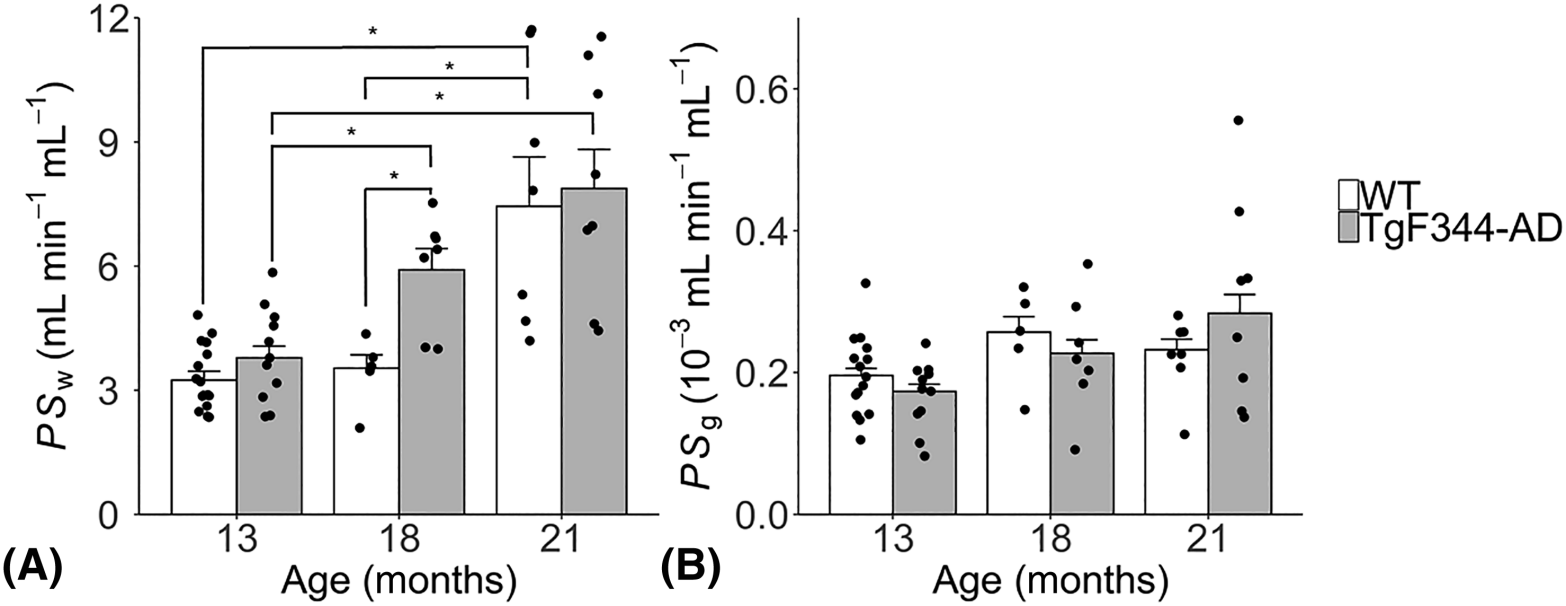
Composite region of interest (ROI) estimates of the permeability surface area product of the blood–brain barrier (BBB) to water, *PS*_w_ (A) and gadolinium‐based contrast agent, *PS*_g_ (B) for TgF344‐AD and wild‐type (WT) rats aged 13, 18 and 21 months. Bar heights show the group means. Points correspond to measurements made in individual rats. Error bars denote standard error of the mean. *statistically significant pairwise comparisons from Tukey post hoc tests (*p* < 0.05)

**FIGURE 4:**
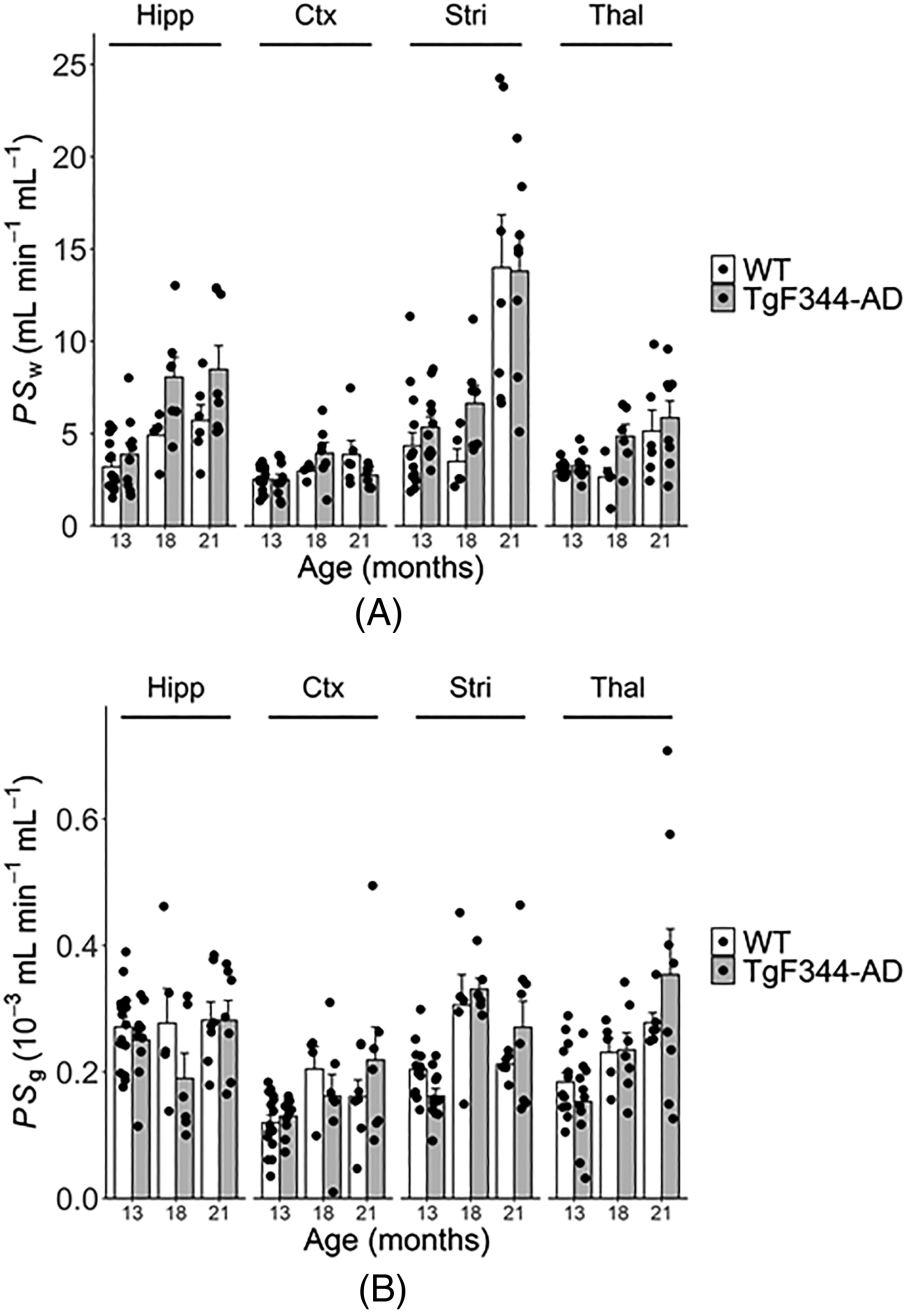
Regional estimates of the permeability surface area product of the blood–brain barrier (BBB) to water, *PS*_w_ (A) and gadolinium‐based contrast agent, *PS*_g_ (B) for TgF344‐AD and wild‐type (WT) rats aged 13, 18 and 21 months. Bar heights show the group means. Points correspond to measurements made in individual rats. Error bars denote standard error of the mean. Ctx, cortex; Hipp, hippocampus; Stri, striatum; Thal, thalamus

**FIGURE 5:**
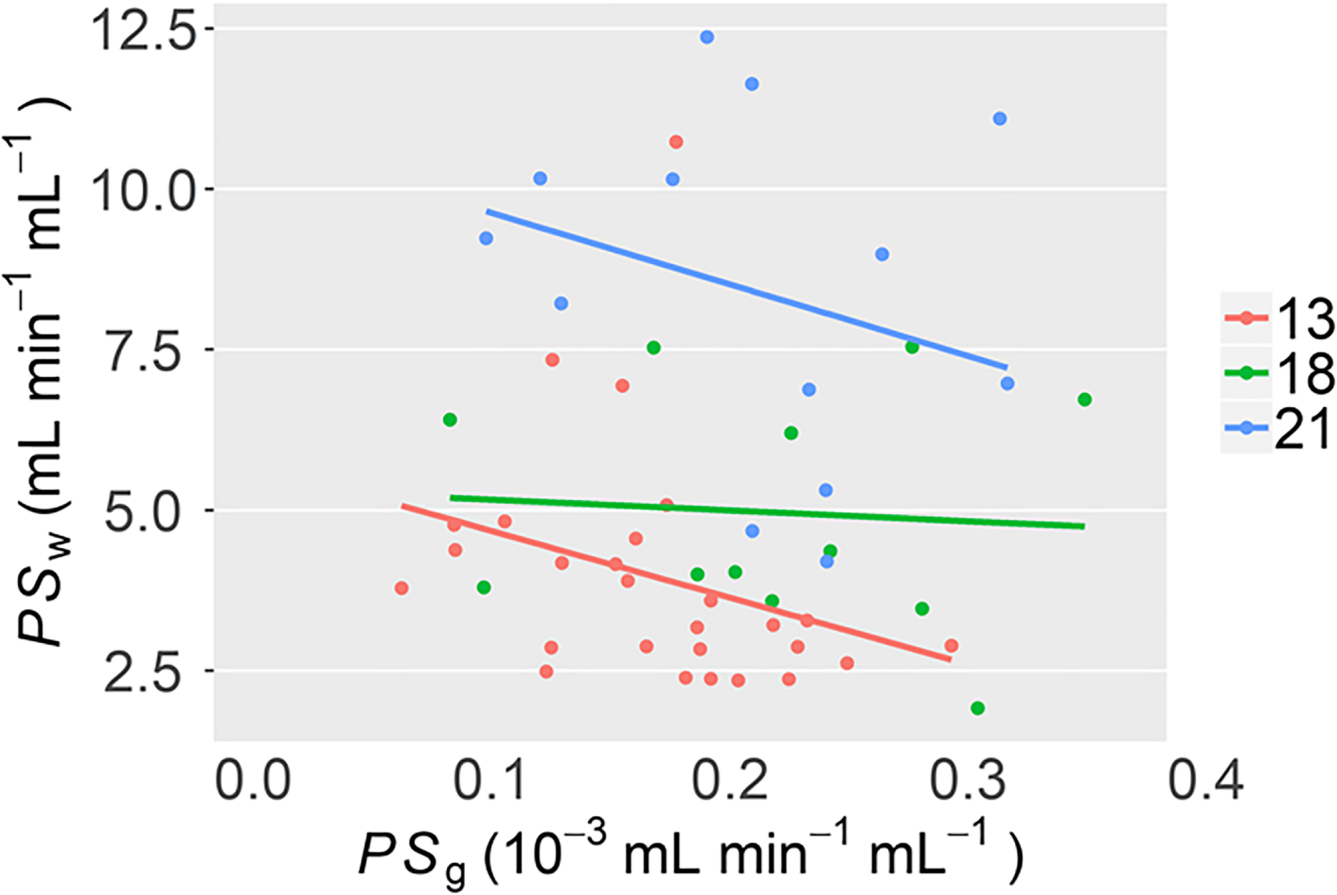
Relationship between composite region of interest (ROI) estimates of *PS*_w_ and *PS*_g_. Regression analysis was applied separately to 13‐, 18‐ and 21‐month data and did not reveal any significant trends between *PS*_w_ and *PS*_g_ (*p* > 0.05). The lack of relationship between *PS*_w_ and *PS*_g_ indicates that in the rat model and age groups studied, blood–brain barrier (BBB) water exchange and BBB leakage of gadoteric acid occur by different mechanisms

Example VIF, ROIs and model fits are shown in Figure 1. Figure 2 shows correlation and Bland–Altman plots for scan‐rescan repeatability measurements. Table 1 shows ROI sizes, volumes, ROI substructures and scan‐rescan CoVs. Figure 3 shows point estimates and group mean values for *PS*_w_ and *PS*_g_ measured in the composite ROI across age and genotype. Tables 2 and 3 show the results of the mixed‐effects model analyses and Tukey post hoc tests for composite ROI *PS*_w_ and *PS*_g_. Figure 4 shows measurements of *PS*_w_ and *PS*_g_ in individual regions (hippocampus, cortex, thalamus and striatum). Tables 4 and 5 show exploratory mixed‐effects model analyses and Tukey post hoc tests for hippocampal *PS*_w_ and *PS*_g_. Figure 5 shows correlation plots between composite ROI estimates of *PS*_w_ and *PS*_g_.

**TABLE 2:** Mixed‐effects model *p‐*values for *PS*_w_ and *PS*_*g*_ in the composite region of interest (ROI)

	*PS*_w_	PS_g_
Age	0.0019	0.22
Genotype	0.0074	0.11
Age × Genotype	0.10	0.28

**TABLE 3:** Percentage difference and post hoc Tukey *p*‐values for genotype and age effects on *PS*_w_ and *PS*_*g*_ in the composite ROI

Genotype effects (WT vs. TgF344‐AD)	*PS*_w_		*PS*_g_	
	% difference	*p*‐value	% difference	*p*‐value
13 months	+14	0.82	−15	0.40
18 months	**+72**	**0.012**	−10	0.99
21 months	+2.9	1.00	+29	0.82
Age effects (WT)				
13m vs. 18m	−4.9	0.99	+10	0.99
18m vs. 21m	**+124**	**0.050**	−11	0.99
13m vs. 21m	**+113**	**0.042**	−2	1.00
Age effects (TgF344‐AD)				
13m vs. 18m‐	**+43**	**0.027**	+17	0.91
18m vs. 21m	+34	0.50	+31	0.71
13m vs. 21m	**+93**	**0.047**	+53	0.21

Positive percentage differences indicate higher values in TgF344‐AD rats (genotype effects) or in older rats (age effects). Negative percentage differences indicate higher values in wild‐types (WTs) (genotype effects) or in younger rats (age effects). Bold indicates significant effects at the 5% level

**TABLE 4:** Mixed‐effects model *p*‐values for *PS*_w_ and *PS*_*g*_ in the hippocampus

	*PS*_w_	*PS*_*g*_
Age	0.0037	0.46
Genotype	0.023	0.28
Age × Genotype	0.25	0.57

**TABLE 5:** Percentage difference and post hoc Tukey *p*‐values for genotype and age effects on *PS*_*w*_ and *PS*_*g*_ in the hippocampus

Genotype effects (WT vs. TgF344‐AD)	*PS*_w_		*PS*_g_	
	% difference	*p*‐value	% difference	*p*‐value
13 months	+21	0.91	−7.6	0.96
18 months	+64	0.22	−32	0.77
21 months	+48	0.59	−0.10	1.0
Age effects (WT)				
13m vs. 18m	+53	0.65	+2.2	1.0
18m vs. 21m	+16	0.99	+1.7	1.0
13m vs. 21m	+78	0.47	+3.9	0.99
Age effects (TgF344‐AD)				
13m vs. 18m	**+108**	**0.0026**	−24	0.81
18m vs. 21m	+5.2	0.99	+48	0.57
13m vs. 21m	**+119**	**0.039**	+12	0.95

Positive percentage differences indicate higher values in TgF344‐AD rats (genotype effects) or in older rats (age effects). Negative percentage differences indicate higher values in wild‐types (WTs) (genotype effects) or in younger rats (age effects). Bold indicates significant effects at the 5% level

### 3.1 Scan‐rescan repeatability of *PS*_w_ and *PS*_g_

The scan‐rescan CoV for the composite ROI was 14% for *PS*_w_ and 8% for *PS*_g_, respectively (Table 1). For individual regions, repeatability of *PS*_w_ and *PS*_g_ was highest in the striatum (CoV_R_ = 13% and 12%, respectively), and lowest in the cortex (CoV_R_ = 38% and 23%, respectively). Figure 2 shows correlation and Bland–Altman plots for scan‐rescan data. Scan 1 and scan 2 estimates of *PS*_w_ and *PS*_g_ had *R* ^2^ values of 0.82 (*p* < 10^−12^) and 0.96 (*p* < 10^−16^), respectively. Bland–Altman analysis showed that 95% of scan‐rescan differences for *PS*_w_ and *PS*_g_ were within ±1.5 and 0.086 x 10^−3^ mL min^−1^ mL^−1^, respectively.

The within‐group variability (incorporating both measurement error and biological variability) of composite ROI *PS*_w_ and *PS*_g_ was 26% and 25%, respectively. Assuming measurement errors and variance due to biological variability add‐in quadrature to give the within‐group variance, biological variance in *PS*_w_ and *PS*_g_ was estimated to be 21% and 23%, respectively. Thus, the within‐group variability was similar for *PS*_w_ and *PS*_g_ and mostly due to biological variation, not random measurement error.

### 3.2 Effects of AD genotype and ageing on *PS*_w_ and *PS*_g_

Mixed‐effects analyses (Table 2) showed significant effects of age (*p* = 0.0019) and genotype (*p* = 0.0074) on composite ROI *PS*_w_, and a borderline genotype × age interaction (*p* = 0.10). Tukey post hoc tests (Table 3) showed no difference in composite ROI *PS*_w_ between TgF344‐AD and WTs at 13 months (*p* = 0.82). Between 13 and 18 months, TgF344‐AD rats exhibited an increase in *PS*_w_ (43% increase; *p* = 0.027), leading to higher *PS*_w_ relative to WTs at 18 months (72% higher; *p* = 0.012), as reported previously.^15^ Between 18 and 21 months, *PS*_w_ increased in WTs (124% increase; *p* = 0.050), but not in TgF344‐AD rats (*p* = 0.50), abolishing the genotype effect observed at the previous time point (Figure 3A). Mixed‐effects analyses showed that there were no effects of age (*p* = 0.22), genotype (*p* = 0.11) or genotype × age (*p* = 0.28) interactions on composite ROI *PS*_g_ (Table 2 and Figure 3B). Post hoc tests also did not show any statistically significant pairwise effects on *PS*_g_ (Table 3).

As an exploratory analysis we also investigated the effects of age and genotype on hippocampal *PS*_w_ and *PS*_g_. Mixed‐effects analyses (Table 4) showed significant effects of age (*p* = 0.0037) and genotype (*p* = 0.023) on hippocampal *PS*_w_, and no genotype × age interaction (*p* = 0.25). Post hoc tests (Table 5) showed significant age effects only in TgF344‐AD rats between 13 and 18 months and between 13 and 21 months. Post hoc tests were unable to detect significant effects of genotype on hippocampal *PS*_w_ at any age. Mixed‐effects analyses and post hoc tests for hippocampal *PS*_g_ showed no significant effects of age or genotype.

Figure 4 shows point estimates and group mean values of *PS*_w_ and *PS*_g_ in the hippocampus, cortex, striatum and thalamus. Qualitatively, the effect of age on *PS*_w_ was largest in the striatum, intermediate in the hippocampus and thalamus, and smallest in the cortex. The effects of genotype at 18 months were of similar magnitude in the hippocampus, striatum and thalamus, and smallest in the cortex. The effect of ageing on *PS*_g_ was also region dependent. *PS*_g_ appeared to increase with age in the cortex and thalamus, was static in hippocampus, and appeared to increase then decrease in the striatum.

Regression analysis showed *PS*_w_ and *PS*_g_ were not linearly related (Figure 5). Coefficient of determination (*R* ^2^) values for 13‐, 18‐ and 21‐month regression lines were all low; *R* ^2^ = (0.09, 0.05, 0.2), and were not statistically significant (*p* = 0.12, 0.82 and 0.060, respectively).

## 4 DISCUSSION

The effects of AD and ageing on the BBB were investigated in TgF344‐AD rats and WTs using MFAME‐DCE MRI. We have previously shown that TgF344‐AD rats exhibit higher BBB permeability to water (*PS*_w_) at 18 months of age compared with WTs. In the same study, we were unable to detect higher leakage of a gadolinium‐based contrast agent (*PS*_g_), indicating that BBB alterations associated with the AD genotype were small, and not of the level that would lead to substantial leakage of blood‐derived proteins into the parenchyma. In this study, we investigated how BBB *PS*_w_ and *PS*_g_ changed with age by scanning at two additional time points: 13 and 21 months. At 13 months of age, we observed no genotype effect in either *PS*_w_ or *PS*_g_, possibly indicating a lack of detectable AD‐related BBB pathology at this time point. Other studies using the same rat model have shown substantial accumulation of parenchymal and capillary amyloid deposition by this age,^27^, ^28^ in addition to reduced vascular reactivity^28^ and functional connectivity.^29^ Despite these changes, the BBB tight junction protein occludin‐1 appears to be maintained.^28^ Between 13 and 18 months, *PS*_w_ increased in TgF344‐AD but not WTs, leading to the significant genotype effect at 18 months, as previously reported. Our previous data showed that changes in *PS*_w_ were correlated with reduced occludin‐1 expression in TgF344‐AD rats relative to WTs.^15^ Between 18 and 21 months, WTs but not TgF344‐AD rats exhibited increases in *PS*_w_, effectively catching up with AD‐related effects that occurred earlier and abolishing the genotype effect observed at 18 months. The lack of genotype effect at 21 months was unexpected and may indicate that the upper limit of detection for measurement of *PS*_w_ was reached. It is known that as the water‐exchange rate across the BBB increases, it becomes experimentally more difficult to measure *PS*_w_ precisely.^30^ This hypothesis is supported by estimates of within‐group variance from our mixed‐effects model analyses, which showed that variance in *PS*_w_ was 2.3 times higher at 21 than at 18 months.

Our results of increased *PS*_w_ with age agree with a recent multi‐TE ASL MRI study conducted by Ohene et al. in young and old C57B1/6JRj mice.^31^ Age‐related reductions in the water‐exchange time (increases in the water‐exchange rate) were accompanied by an increase in aquaporin‐4 mRNA expression but a decrease in α‐synotrophin mRNA, a protein responsible for anchoring aquaporin‐4 to astrocyte end feet. Unfortunately, other factors affecting BBB integrity such as tight junction expression were not assessed, making it difficult to interpret the underlying cause of increased water permeability. Furthermore, the changes in *PS*_w_ observed in our study are much larger than in the study by Ohene et al.^31^ (113% between 13 and 21 months vs. 32% between 7 and 27 months). The reason for this difference is not clear but may be due to differences in water exchange between species, or it could reflect differences in the MRI method used to quantify water exchange.

We did not observe effects of age or genotype on *PS*_g_. The literature on the effects of AD on BBB leakage of MRI gadolinium‐based contrast agents and other ‘non‐essential’ molecules is contradictory; while recent studies have demonstrated an increase in BBB leakage, a similar number of studies have failed to detect changes in rodent models of AD^32^, ^33^ and human disease.^19^, ^34^ Those studies that have detected increased leakage of gadolinium contrast agents report group‐level effects that are much smaller than the within‐group variability,^12^ and thus likely only detectable using very large sample sizes. In this study, measurement repeatability was similar for *PS*_w_ and *PS*_g_, and it is possible that we were able to detect differences in *PS*_w_ but not *PS*_g_ because water is a much smaller molecule and more likely to be affected by finer scale alterations to BBB function.

To determine whether the degree of BBB water‐exchange was related to the degree of BBB gadolinium leakage, we correlated estimates of *PS*_w_ and *PS*_g_ made in the same animal. We did not find evidence of a relationship between the two parameters. In healthy brain, it may be expected that these parameters are unrelated, because their transport across the BBB is governed by different mechanisms.^30^ A lack of correlation may also be expected if BBB changes affect one measure but not the other. We know from our previous work that the tight junction protein occludin‐1 is affected from 18 months in this rat model. If these changes were sufficient, it could be expected that *PS*_w_ and *PS*_g_ may be related, since para‐cellular diffusion would dominate for both water and contrast agent. However, if these changes were too small to affect the leakage of gadolinium, then *PS*_w_ and *PS*_g_ are still unlikely to be related. Future work should investigate the limits of sensitivity of water‐exchange measurements, and determine under which conditions, if any, *PS*_w_ and *PS*_g_ are related.

The current study has the following limitations. Measurement of both *PS*_g_ and *PS*_w_ within a single examination meant that the protocol for determining *PS*_g_ was suboptimal. Compared with a standard DCE‐MRI protocol, data between the first pass peak and tail of the gadolinium washout curves were missing, as this time was used to acquire multiflip angle data for *PS*_w_ estimation. The lack of data during this period may have increased variability in *PS*_g_ estimates, making group differences in *PS*_g_ more difficult to detect experimentally. Further work should determine if *PS*_g_ obtained using a standard (full data) DCE‐MRI protocol can detect age‐ and AD‐related BBB alterations in this model. The groups studied at 13 and 21 months had an uneven mix of males and females. It is not known whether there are gender differences in BBB integrity in this model. If present, this may have limited our ability to detect genotype effects at 13 and 21 months. A major limitation is the lack of confirmatory immunohistochemistry, such as staining for tight junction proteins and aquaporin‐4. Unfortunately, while the study team extracted brains from all animals (see the Materials and Methods section), tissue was damaged in storage and did not produce satisfactory staining results. This limits our ability to understand the microscopic BBB changes governing the observed changes in water permeability. Finally, the small sample size, particularly for the 18‐ and 21‐month‐old groups, meant that statistical power was too low to model and test variability in *PS*_w_ and *PS*_g_ across multiple different regions. Regional estimates of *PS*_w_ and *PS*_g_ were generated in the hippocampus, cortex, thalamus and striatum, but the variation in *PS*_w_ and *PS*_g_ across regions was not formally evaluated in statistical models or post hoc tests. It is known that AD pathologies vary in severity across the brain and the use of a composite ROI may have masked regional differences in BBB pathology. We therefore performed an exploratory analysis to determine *PS*_w_ and *PS*_g_ alterations in the hippocampus, a region known to be affected early in AD.^4^ This analysis showed changes in *PS*_w_ between 13 and 18 months and between 13 and 21 months in TgF344‐AD rats only. In contrast to the composite ROI, post hoc tests showed no genotype effects at any time point. In agreement with the composite ROI, no changes in *PS*_g_ were observed with age or genotype. The lack of hippocampal genotype and WT age effects on *PS*_w_ are likely because of the absence of striatal and thalamic contributions, which appear to strongly contribute towards these effects (Figure 4), but could also be due to the lower repeatability of hippocampal measurement due to smaller ROI size (Table 1).

In conclusion, we have used MFAME‐DCE MRI to investigate the effects of age and AD on BBB permeability surface area products of water and a gadolinium‐based contrast agent. We observed increases in BBB *PS*_w_ with age in both TgF344‐AD and WT rats, and found that these changes occurred earlier in TgF344‐AD rats. These results indicate that AD pathology may accelerate the onset of BBB breakdown that occurs as part of the normal ageing process. Further work is needed to understand the complex structural changes occurring at the BBB that account for the alterations to *PS*_w_ observed in this study.

## Supporting information


**Data S1.** Supporting information
