# Supplementary material for: Alzheimer's disease pathology is associated with earlier alterations to blood–brain barrier water permeability compared with healthy ageing in TgF344‐AD rats
Source: NMR Biomed. 2021 Mar 15;34(7):e4510. doi: 10.1002/nbm.4510 (PMC11475392; doi:10.1002/nbm.4510)
Supplement: Supplementary file 1 — Data S1. Supporting information [file NBM-34-e4510-s001.docx]

**SUPPLEMENTARY MATERIALS**

**Animal breeding, housing, and husbandry**

Throughout their lifetime, animals were housed in a conventional rodent facility in individually ventilated cages (between 3-4 animals per cage). The facility had a 12/12 hour dawn/dusk cycle, beginning at 7.30 am. Bedding was aspen woodchip and paper sizzle nest. Animals were given the following environmental enrichment: sizzle nest, play tunnels, aspen bricks (chew sticks) and aspen balls. Housing temperature and humidity were maintained at 19-23°C and 45-65% respectively, conforming to the Home Office code of practice for housing rodents. All animals were fed ad libitum on the BK001(E) diet (standard diet) and given free access to water. Animal health was checked daily and respiratory problems assessed prior to anaesthesia. After MRI, animals were recovered until they were able to take food and water, then returned to housing.
